# Activity-difference maps and consensus similarity measure characterize structure-activity relationships

**DOI:** 10.1186/1758-2946-4-S1-P24

**Published:** 2012-05-01

**Authors:** José L Medina-Franco, Austin B Yongye, Jaime Pérez-Villanueva, Richard A Houghten, Karina Martínez-Mayorga

**Affiliations:** 1 Torrey Pines Institute for Molecular Studies, Port St. Lucie, Florida, 34987, USA; 2 Departament of Biological Systems, UAM-X, Mexico City, 04960, Mexico

## 

Dual and triple activity-difference (DAD/TAD) maps are two- and three- dimensional representations of the pairwise activity differences of compound data sets, respectively [1]. These maps are valuable tools for the systematic characterization of structure-activity relationships (SAR) of compounds data sets screened against two or three targets [2]. Adding pairwise structural similarity information into the DAD/TAD maps readily reveals *activity cliff*[3] regions in the SAR for one, two or the three targets. In addition, pairs of compounds in the smooth regions of the SAR and scaffold hops are also easily identified in these maps. Herein, we describe DAD and TAD maps for the systematic characterization of the SAR of data sets screened against three molecular targets. Several 2D and 3D structure representations were used to characterize the SAR in order to reduce the well-known dependence of the activity landscape on the structural representation [4,5]. Systematic analysis of the DAD and TAD maps reveals regions in the landscape with similar SAR for two or the tree targets as well as regions with inverse SAR, i.e., changes in structure that increase activity for one target, but decrease activity for the other target. Focusing the analysis on pairs of compounds with high structure similarity revealed the presence of single-, dual- and triple-target activity cliffs, i.e., small changes in structure with high changes in potency for one, two or the three targets, respectively. Triple-target scaffold hops are also discussed.

## References

[B1] Pérez-Villanueva J Santos R Hernández-Campos A Giulianotti MA Castillo R Medina-Franco JL Structure-Activity Relationships of Benzimidazole Derivatives as Antiparasitic Agents: Dual Activity-Difference (DAD) Maps Med Chem Comm 2011 2 44 49 10.1039/c0md00159g

[B2] Dimova D Wawer M Wassermann AM Bajorath J Design of Multitarget Activity Landscapes that Capture Hierarchical Activity Cliff Distributions J Chem Inf Model 2011 51 258 266 10.1021/ci100477m 21275393

[B3] Maggiora GM On Outliers and Activity Cliffs-Why QSAR Often Disappoints J Chem Inf Model 2006 46 1535 1535 10.1021/ci060117s 16859285

[B4] Medina-Franco JL Martínez-Mayorga K Bender A Marín RM Giulianotti MA Pinilla C Houghten RA Characterization of Activity Landscapes Using 2D and 3D Similarity Methods: Consensus Activity Cliffs J Chem Inf Model 2009 49 477 491 10.1021/ci800379q 19434846

[B5] Yongye A Byler K Santos R Martínez-Mayorga K Maggiora GM Medina-Franco JL Consensus Models of Activity Landscapes with Multiple Chemical, Conformer and Property Representations J Chem Inf Model 2011 51 1259 1270 10.1021/ci200081k 21609014

